# Prognostic role of pre-procedural lung ultrasound in patients undergoing transcatheter aortic valve implantation

**DOI:** 10.3389/fcvm.2025.1626497

**Published:** 2025-07-29

**Authors:** Guilherme Pinheiro Machado, Pedro Castilhos de Freitas Crivelaro, Gustavo Neves de Araujo, Guilherme Heiden Telo, Luiz Carlos Corsetti Bergoli, Marina Petersen Saadi, Julia Carvalho da Silva, Camila Porto Cardoso, Wagner Azevedo, Felipe Costa Fuchs, Marco Wainstein

**Affiliations:** ^1^ Cardiology Department, Hospital de Clínicas de Porto Alegre, Porto Alegre, Brazil; ^2^ Cardiology Department, Hospital da Unimed Grande Florianópolis, São José, Brazil; ^3^ Faculty of Medicine, Universidade Federal do Rio Grande do Sul, Porto Alegre, Brazil; ^4^ Faculty of Medicine, Universidade Federal de Ciências de Saúde de Porto Alegre, Porto Alegre, Brazil

**Keywords:** transcatheter aortic valve replacement, all-cause mortality, aortic stenosis, lung ultrasound (LUS), heart failure

## Abstract

**Background:**

Transcatheter aortic valve implantation (TAVI) is the preferred treatment for patients with severe aortic stenosis (AS) in patients >75 years. Lung ultrasound (LUS) has emerged as a noninvasive tool for assessing pulmonary congestion and risk stratification in cardiovascular disease, especially heart failure, however, its prognostic role in TAVI remains to be clarified. Therefore, our aim was to investigate the association between pre-procedural LUS findings and clinical outcomes in patients undergoing TAVI.

**Methods:**

We conducted a prospective cohort study of 116 patients undergoing TAVI from 2021 to 2024. Lung ultrasound was performed immediately before the procedure. Patients were classified as having “wet lungs” (≥1 positive zone) or “dry lungs” (no positive zones). Exclusion criteria were lack of consent or absence of pre-procedural ultrasound assessment. The primary endpoint was a composite of all-cause mortality and heart failure hospitalization within 12 months. Secondary endpoints included VARC-2-defined complications.

**Results:**

Among 85 patients included in the final analysis the mean age was 80 ± 11 years, 51.8% were male, and 55 (64.7%) had wet lungs. Patients with wet lungs had higher STS-PROM scores (5.2% vs. 3.0%, *p* < 0.001), but there were no significant differences in the primary outcome (3.8% vs. 6.7%, *p* = 0.55). Moreover, procedural characteristics and complication rates were similar between groups.

**Conclusions:**

Pre-procedural LUS was not associated with worse outcomes following TAVI. While LUS may reflect comorbidity burden, its isolated prognostic value in this setting appears limited.

## Graphical Abstract

**Figure:**
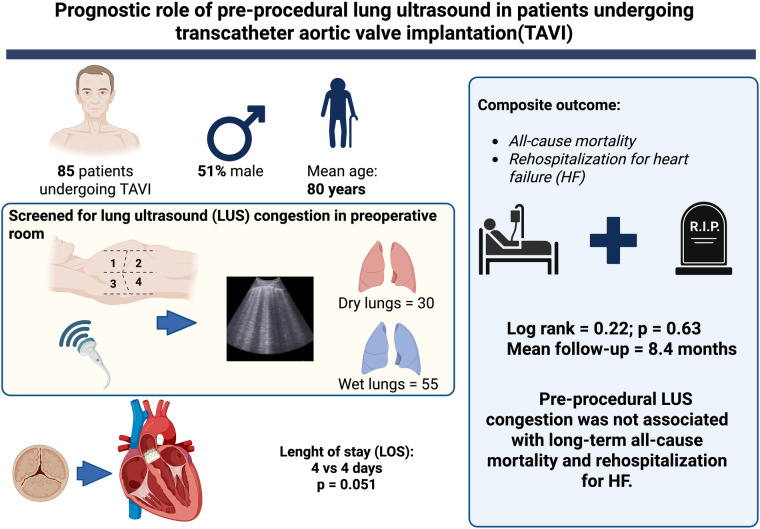
Central illustration – Pre-procedural lung ultrasound in patients undergoing transcatheter aortic valve implantation (TAVI). Figure Created with biorender.com.

## Introduction

Aortic stenosis (AS) is a prevalent valvular disease among the elderly with an increasing incidence due to the aging of the population. Disease progression and onset of symptoms carry significant morbidity and impact on patients' lives (1). The treatment inevitably requires replacement of the aortic valve, which can be done surgically or, more recently, percutaneously (2–4). The development of heart failure (HF) symptoms is a key determinant of survival in patients with aortic stenosis. The progressive increase in left ventricular diastolic pressure leads to pulmonary congestion and dyspnea, significantly impacting patient prognosis. Even in patients who have undergone transcatheter aortic valve implantation (TAVI), HF is the most common cause of cardiac rehospitalization (5).

Lung ultrasound (LUS) is a well-established, noninvasive imaging modality that enhances clinical assessment by providing real-time evaluation of pulmonary congestion. It has demonstrated high sensitivity in detecting lung congestion and has been shown to predict adverse outcomes in patients with ST-elevation myocardial infarction (STEMI), cardiogenic shock, and HF (6–10). The 2021 ESC Guidelines for acute and chronic HF recommend echocardiography for cardiac assessment, while LUS is the only non-cardiac ultrasound method recommended at admission, during hospitalization, and at discharge (11).

In patients with AS, the presence of lung congestion assessed by LUS has been associated with an increased risk of cardiovascular events (12). However, evidence on the prognostic value of LUS in patients undergoing TAVI population remains limited. To date, only one study has assessed its role reporting that lung congestion was independently associated with cardiovascular events (13). Nonetheless, in this study, lung ultrasound was performed the day before the procedure, and as lung congestion can change in few hours, the results may be biased. Moreover, it was conducted at a single center with a small sample size, and its findings require validation in larger cohorts. Therefore, this study aims to investigate the role of LUS performed immediately before TAVI and to evaluate its association with cardiovascular outcomes up to 12 months follow-up.

## Methods

### Data, study design and population

This prospective study was conducted in a tertiary university hospital in Southern Brazil between 2021 and 2024. Patients eligible for inclusion were consecutive adults (≥18 years of age) with indication of aortic valve replacement, based on the presence of severe aortic stenosis characterized by the following criteria: (1) ejection flow velocity greater than 4 m/s; (2) a mean transvalvular aortic pressure gradient of at least 40 mmHg in the presence of normal flow; or (3) an effective aortic orifice no larger than 1.0 cm (3, 4). Patients with low-flow, low-gradient aortic stenosis with reduced ejection fraction (mean gradient <40 mmHg, valve area ≤1 cm^2^, LVEF <50%), previous surgical aortic prothesis dysfunction or pure aortic insufficiency submitted to TAVI were also included. Patients were also categorized based on the classification proposed by Généreux et al. (14) on the extent of cardiac damage: no cardiac damage (Stage 0), LV damage (Stage 1), LA or mitral valve damage (Stage 2), pulmonary vasculature or tricuspid valve damage (Stage 3), or right ventricular damage (Stage 4). All patients provided written informed consent. This study was approved by the Institutional Research Ethics Committee. Manuscript writing was guided according to STROBE guideline for reporting observational studies (15).

TAVI procedures were performed according to current standard techniques. Data from medical records were prospectively acquired and transferred to standardized case report forms (CRFs). The following variables were collected: baseline clinical characteristics, medical history, procedure characteristics, pharmacological treatment in intensive care unit, need for hemodynamics monitoring devices and discharge therapies. Long-term follow-up was conducted through outpatient visits. Study data were transferred and managed using REDCap electronic data capture tools hosted at Hospital de Clínicas de Porto Alegre.

### Ultrasound assessment

A portable ultrasound machine (Mindray, Shenzhen Mindray Bio-medical Electronics Co., Ltd., model MT3) equipped with a 2.5-MHz sectorial transducer was used (16). Ultrasound assessment was performed immediately before procedure at preoperative room. Two trained investigators with ultrasound expertise (GPM & MS, interventional cardiology and echocardiography fellows) performed all echocardiographic examinations. The examination consisted of bilateral scanning of the anterior and lateral chest wall, with sagittal (i.e., perpendicular to the intercostal space) probe orientation and the patient in the supine position. The chest wall was divided into eight zones, and one scan for each zone was obtained. The zones were two anterior and two lateral per side (Central Illustration). A zone was considered positive if it had at least three B-lines (9). The acquisition of lung ultrasound images requires positioning the transducer perpendicular to the chest wall within the intercostal spaces to avoid rib shadowing to ensures optimal visualization of the pleural line and the lung parenchyma below. The depth settings are adjusted to clearly display the pleura and the underlying lung tissue. B-lines, which are vertical artifacts, typically extend from the pleural line to the bottom of the screen, obscuring A-lines and moving synchronously with the patient's breath (17). Image acquisition and analysis was performed on-site, with immediate upload into the study dataset. Investigators performed a pre-study with 23 patients in CICU to assess interobserver variability with excellent agreement observed for lung positive zone assessments (ICC, 0.903; 95% CI, 0.818–0.954; *p* < 0.001).

### Outcomes

The primary endpoint was composite endpoint (all-cause mortality or re-hospitalization for HF. All complications and study end-points were pre-specified to be reported at the beginning of the registry according to the Valve Academic Research Consortium-2 (VARC-2) criteria (18), including death from any cause, stroke, life-threatening or disabling bleeding, major vascular complication, stage 2 or 3 acute renal failure, coronary obstruction requiring intervention or surgery, and new percutaneous or surgical procedure to correct valve dysfunction. Length of stay in a critical unit, length of stay, need for re-hospitalization and mortality up to 12 months were also evaluated. Heart failure hospitalization was defined as patient's hospital admission with a primary diagnosis of HF with a length of stay in in hospital for at least 24 h.

### Statistical analysis

Continuous variables were expressed as mean ± standard deviation (SD) or median (interquartile range), according to data normality. The normality of the distribution of each variable was assessed by the Shapiro–Wilk test. Categorical variables were expressed as relative and absolute frequencies. Patient groups were compared using independent samples Student's *t*-test (for normally distributed variable) or Mann- Whitney *U*-test (for other variables) for continuous variables and χ^2^ test or Fisher's exact tests for categorical variables. All statistical analyses were conducted SPSS Statistics for Windows, Version 29.0. (IBM Corp., Armonk, NY). All hypothesis tests had a two-sided significance *p* level of 0.05.

## Results

A total of 116 patients submitted to TAVI performed at Hospital de Clínicas de Porto Alegre, Brazil, from July 2021 to December 2024 and 85 were included in the final analysis. 31 patients were excluded due to lack of consent or absence of pre-procedural ultrasound assessment. Among patients included in the registry, 84 (98.8%) had severe native aortic stenosis and 1 (1.2%) had a degenerated bioprosthetic surgical valve. Mean age was 80 years (±11), and 44 (51.8%) were male. Mean STS predicted risk of mortality (STS-PROM) score was 4.4% (±3.0). Mean aortic valve area was 0.71 (±0.16) cm^2^ with a mean transvalvular gradient of 46 (±13) mmHg. Baseline characteristics of the patients are best summarized in Table 1.

**Table 1:** Demographic characteristics.

Demographic characteristics	Overall *n* = 85	Dry lungs *n* = 30	Wet lungs *n* = 55	*p*-value
Age, years	80 (±11)	77.8 (±16)	82.3(±6.1)	0.07
BMI, kg/m^2^	26.6 (±4.9)	27.2(±5.8)	26.3(±4.4)	0.42
Male sex	44 (51.8)	19 (63.3)	25 (45.5)	0.11
Hypertension	70 (83.3)	24 (80)	46 (85.2)	0.54
Diabetes	26 (31.0)	10 (33)	16 (29.6)	0.72
Previous MI	8 (9.5)	4 (13.3)	4 (7.4)	0.37
Previous stroke	5 (6.0)	4 (13.3)	1 (1.9)	0.03
COPD	12 (14.3)	6 (20)	6 (11.1)	0.26
CKD	17 (20.2)	6 (20.0)	11 (20.4)	0.96
Atrial fibrillation	20 (23.8)	6 (20)	14 (25.9)	0.54
Prior RBBB	8 (9.4)	1 (3.3%)	7 (12.7)	0.15
Syncope	19 (22.6)	8 (26)	11 (20.4)	0.50
STS-PROM score (%)	4.4 (± 3.0)	3.0 (±1.4)	5.2 (±3.4)	<0.001
LVEF (%)	57 (±14.3)	58.2 (15.4)	56.9 (13.8)	0.71
Valvar area, cm^2^	0.71 (±0.16)	0.73 (±0.18)	0.70 (0.15)	0.48
Mean gradient, mmHg	46.7 (±13.1)	46.4(±15.7)	46.8 (±11.5)	0.90
*E/e’*	17.0 (±5.0)	14.8 (±3.8)	18.6 (±5.2)	0.09
TAPSE, mm	20.1 (±6.6)	23.0 (±10.1)	18.7(±3.6)	0.06
PSAP, mmHg	40 (±15.8)	38.4(±13.0)	42.2(±17.3)	0.37
Moderate or severe MR	11 (12.9)	5 (16.7)	6 (10.9)	0.45
Moderate or severe TR	5 (5.9)	0 (0)	5 (9.1)	0.89
LVMi	108.9 (±29.2)	99.6 (23)	114.2 (30.8)	0.41
LVAi	47.7 (±13.0)	44.6 (9.6)	49.5 (14.3)	0.11
Généreux stage III or IV	12 (14.1)	2 (6.7)	10 (18.2)	0.14

Values are expressed as mean ± standard deviation, median (interquartile range) or number (%.). BMI, body mass index; MI, myocardial infarction; RBBB, right bundle branch block; COPD, chronic obstructive pulmonary disease; CKD, chronic kidney disease; LVEF, left ventricle ejection fraction; STS-PROM, Society of Thoracic Surgeons predicted risk of mortality; TAPSE, tricuspid annular plane systolic excursion; E/e’, ratio of early diastolic filling/tissue Doppler velocity annulus; PASP, pulmonary artery systolic pressure; MR, mitral regurgitation; TR, tricuspid regurgitation; LVMi, left ventricular mass index; LVAi, left atrial volume index. Source: Authors.

Procedural data are described in Table 2. Conscious sedation was performed in 74 (87%) Transfemoral access was used in 83 (97.6%) cases. Most common prosthesis were Sapien 3 and Evolut Pro in 34 (40%) and 25 (29.4%) cases, respectively. There was no difference in contrast volume and procedural time.

**Table 2:** Procedural characteristics.

Procedural characteristics	Overall *n* = 85	Dry lungs *n* = 30	Wet Lungs *n* = 55	*p*-value
Conscious sedation	74 (87.1)	27 (90)	47 (85.5)	0.55
Transfemoral approach	83 (97.6)	30 (100)	53 (96.4)	0.57
Prothesis				
Evolut R	8 (9.4)	0 (0)	8 (14.5)	0.007
Evolut Pro	25 (29.4)	10 (33.3)	15 (27.3)	
Evolut Pro+	15 (17.6)	2 (6.7)	13 (23.6)	
Sapien 3	34 (40.0)	18 (60.0)	16 (29.1)	
Other	1 (3.5)	0 (0)	3 (5.5)	
Size				
20 mm	1 (1.2)	0 (0)	1 (1.2)	0.27
23 mm	19 (22.6)	9 (30.0)	10 (18.5)	
26 mm	23 (27.4)	7 (23.3)	16 (29.6)	
29 mm	34 (40.5)	14 (46.7)	20 (37.0)	
34 mm	6 (7.1)	0 (0)	6 (11.1)	
Procedure time, min	114.7 (±40.7)	107.0 (±25.7)	118.3 (±46.0)	0.36
Contrast volume, ml	139.3 (±47.8)	134.7 (±45.6)	141 (±49.1)	0.57

Values are expressed as mean ± standard deviation, median (interquartile range) or number (%.).

* Source: Authors.

The primary outcome occurred in 4 patients (4.8%) with no difference between wet and dry lungs (Graphical Abstract - Central Illustration). Device success was achieved in 82 patients (96.5%). Procedural related death and in-hospital mortality were 2 (2.4%) and 1 (1.2%), respectively. New permanent pacemaker was required in 13 (15.3%). Median follow-up was 8.4 months (2.6–15.4). Other outcomes are described in Table 3.

**Table 3:** Clinical outcomes.

Clinical Outcomes	Overall *n* = 85	Dry lungs *n* = 30	Wet lungs *n* = 55	*p*-value
Primary Outcome				
Composite outcome	4 (4.8)	2 (6.7)	2 (3.8)	0.55
All cause death	3 (3.6%)	1 (3.3)	2 (3.8)	0.91
Rehospitalization for HF	3 (3.8)	2 (6.7)	1 (1.9)	0.26
Secondary Outcomes				
Device success	82 (96.5)	30 (100)	52 (94.5)	0.19
Procedure related death	2 (2.4)	0 (0)	2 (3.6)	0.29
In-hospital death	1 (1.2)	0 (0)	1 (1.8)	0.45
Major vascular complication	3 (5.9)	3 (10.0)	2 (3.6)	0.23
Major bleeding	2 (2.4)	1 (3.3)	1 (1.9)	0.66
Life-threatening bleeding	1 (1.2)	0 (0)	1 (1.8)	0.45
All stroke	1 (1.2)	0 (0)	1 (1.8)	0.45
Acute kidney injury	5 (5.9)	1 (3.3)	4 (7.3)	0.46
New-onset LBBB	18 (21.2)	6 (20.0)	12 (21.8)	0.84
Conduction disturbance	31 (36.5)	8 (26.7)	23 (41.8)	0.16
New permanent pacemaker	13 (15.3)	3 (10)	10 (18.2)	0.31
ICU, days	1 (1–2)	1 (1–2)	2 (2–3)	0.14
Length of stay after procedure, days	4 (2.2–6.7)	4 (2–7)	4 (3–6.2)	0.51
Early safety at 30 days	72 (84.7.)	27 (90)	45 (81.8)	0.31
Valve related dysfunction	2 (2.5)	1 (3.4)	1 (2.0)	0.68
Clinical efficacy after 30 days	72 (87.8)	27 (90)	45 (86.5)	0.64
Median follow-up, months	8.4 (2.6–15.4)	8.5 (4.4–17.1)	7.5 (1.5–14.2)	0.63
LVEF, %	59 (±10.1)	57.7 (11)	61.9 (8)	0.30

Values are expressed as mean ± standard deviation, median (interquartile range) or number (%.). HF, heart failure; LBBB, left bundle branch block; ICU, intensive care unit; LVEF, left ventricle ejection fraction. Source: Authors.

## Discussion

This study evaluated the prognostic utility of LUS performed immediately before TAVI in a cohort of patients. Despite recommendation by the ESC guidelines (11) in acute and chronic HF of performing LUS at admission, during hospitalization, and at discharge in patients with HF, pre-procedural LUS was not associated with worse clinical outcomes, including mortality and heart failure hospitalization within 12 months.

Pulmonary congestion detected by lung ultrasound reflects elevated left-sided filling pressures and has been established as a powerful prognostic marker in heart failure (7, 19, 20) and acute coronary syndromes (8–10, 21, 22), as LUS can outperform physical examination in detecting subclinical congestion (16). However, its prognostic relevance in patients undergoing TAVI remains largely unexplored.

Pulmonary congestion is commonly present in patients with severe aortic stenosis, especially in advanced stages of the disease (12). However, data regarding the prognostic relevance in the TAVI population remains sparse. Lillo et al. (13) demonstrated that pre-procedural lung congestion assessed by LUS was independently associated with increased cardiovascular events (composite of all-cause mortality, hospitalization for heart failure and urgent medical visits for worsening dyspnea) in TAVI patients at 12-month follow-up. These discrepancies with our findings may be explained by differences in methodology. In the present study, patients underwent LUS immediately prior to the procedure, minimizing potential confounding from dynamic fluid shifts and reflecting a real-time physiologic snapshot. This approach aimed to minimize variability and capture the patient's true baseline hemodynamic state. In contrast, Lillo et al. assessed LUS the day before TAVI, potentially allowing for clinical changes to occur in the interim, especially if medical interventions were performed based on this information. Interestingly, although patients with pulmonary congestion detected by lung ultrasound had higher STS-PROM scores and a trend toward more advanced cardiac damage stages, clinical outcomes did not significantly differ. This finding suggests that correction of valve obstruction and prompt hemodynamic relief – reducing left ventricular afterload and improving pulmonary pressures - may attenuate the impact of pre-procedural pulmonary congestion on short- and mid-term prognosis.

From a clinical perspective, LUS may serve as a marker of overall disease burden or frailty but does not necessarily provide incremental prognostic value over established risk scores in TAVI candidates. Additionally, it is possible that other confounders, such as optimization of perioperative care and fluid status management, may mitigate the effects of pre-existing pulmonary congestion.

Our findings highlight that although LUS is a valuable bedside tool in several scenarios, its role in prognostic stratification before TAVI may be limited when used in isolation. It may be more useful when integrated into a multimodal risk assessment strategy that includes echocardiographic parameters, biomarkers, and functional assessment. However, it is important to note that the limited sample size reduces the precision of our estimates and the ability to detect statistically significant associations, especially for less frequent outcomes. This limitation should be considered when interpreting the findings.

### Strengths and limitations

This study has several strengths, including prospective data collection, standardized ultrasound assessment, and follow-up within a real-world public health setting. However, limitations must be acknowledged. The single center and small sample size may have been underpowered to detect small differences in outcomes, additionally, and to detect associations with rare outcomes. Given the limited number of events it was not possible to perform a reliable multivariable analysis without risking model overfitting. LUS was performed by trained investigators, but images were not reviewed by an independent core laboratory which may introduce observer bias. Additionally, although LUS was conducted immediately before the procedure, the single time-point assessment may not reflect dynamic pulmonary congestion status throughout hospitalization.

## Conclusions

Pre-procedural lung ultrasound was not associated with worse outcomes after TAVI. While LUS remains a valuable tool for bedside assessment of pulmonary congestion, its isolated prognostic value in this population appears limited. Future studies should explore its role in combination with other clinical and hemodynamic parameters and in larger multicenter cohorts.

## Data Availability

The data that support the findings of this study are available from the corresponding author, upon reasonable request. The data are not publicly available due to containing information that could compromise the privacy of research participants.

## References

[1] Nkomo VT Gardin JM Skelton TN Gottdiener JS Scott CG Enriquez-Sarano M. Burden of valvular heart diseases: a population-based study. Lancet. (2006) 368(9540):1005–11. 10.1016/S0140-6736(06)69208-8 16980116

[2] Cribier A Eltchaninoff H Bash A Borenstein N Tron C Bauer F Percutaneous transcatheter implantation of an aortic valve prosthesis for calcific aortic stenosis. Circulation. (2002) 106(24):3006–8. 10.1161/01.CIR.0000047200.36165.B8 12473543

[3] Vahanian A Beyersdorf F Praz F Milojevic M Baldus S Bauersachs J 2021 ESC/EACTS guidelines for the management of valvular heart disease: developed by the task force for the management of valvular heart disease of the European Society of Cardiology (ESC) and the European association for cardio-thoracic surgery (EACTS). Eur Heart J. (2022) 43(7):561–632. 10.1093/eurheartj/ehab395 34453165

[4] Otto CM Nishimura RA Bonow RO Carabello BA Erwin JP Gentile F 2020 ACC/AHA guideline for the management of patients with valvular heart disease: a report of the American College of Cardiology/American Heart Association joint committee on clinical practice guidelines. Circulation. (2021) 143(5):e72–227. 10.1161/CIR.0000000000000923 33332150

[5] Vemulapalli S Dai D Hammill BG Baron SJ Cohen DJ Mack MJ Hospital resource utilization before and after transcatheter aortic valve replacement: the STS/ACC TVT registry. J Am Coll Cardiol. (2019) 73(10):1135–46. 10.1016/j.jacc.2018.12.049 30871697

[6] Öhman J Harjola V-P Karjalainen P Lassus J. Rapid cardiothoracic ultrasound protocol for diagnosis of acute heart failure in the emergency department. Eur J Emerg Med. (2019) 26(2):112–7. 10.1097/MEJ.0000000000000499 28984662

[7] Rivas-Lasarte M Álvarez-García J Fernández-Martínez J Maestro A López-López L Solé-González E Lung ultrasound-guided treatment in ambulatory patients with heart failure: a randomized controlled clinical trial (LUS-HF study). Eur J Heart Fail. (2019) 21(12):1605–13. 10.1002/ejhf.1604 31667987

[8] Scolari FL Machado GP Pagnoncelli A Chies A de Araujo GN da Silveira AD Lung ultrasound evaluation of SCAI shock stages predicts mortality in ST-segment elevation myocardial infarction. JACC Cardiovasc Imaging. (2023) 16(2):260–2. 10.1016/j.jcmg.2022.09.005 36648045

[9] Araujo GN Silveira AD Scolari FL Custodio JL Marques FP Beltrame R Admission bedside lung ultrasound reclassifies mortality prediction in patients with ST-segment-elevation myocardial infarction. Circ Cardiovasc Imaging. (2020) 13(6):e010269. 10.1161/CIRCIMAGING.119.010269 32536197

[10] Machado GP Telo GH de Araujo GN da Rosa Barbato JP Amon A Martins A A combination of left ventricular outflow tract velocity time integral and lung ultrasound to predict mortality in ST elevation myocardial infarction. Intern Emerg Med. (2024) 19(8):2167–76. 10.1007/s11739-024-03719-z 39044051

[11] McDonagh TA Metra M Adamo M Gardner RS Baumbach A Böhm M 2021 ESC guidelines for the diagnosis and treatment of acute and chronic heart failure: developed by the task force for the diagnosis and treatment of acute and chronic heart failure of the European Society of Cardiology (ESC) with the special contribution of the heart failure association (HFA) of the ESC. Eur Heart J. (2021) 42(36):3599–726. 10.1093/eurheartj/ehab368 34447992

[12] Szabó IA Gargani L Morvai-Illés B Polestyuk-Németh N Frigy A Varga A Prognostic value of lung ultrasound in aortic stenosis. Front Physiol. (2022) 13:1–8. 10.3389/fphys.2022.838479 PMC9037236 35480045

[13] Lillo R Cangemi S Graziani F Locorotondo G Pedicino D Aurigemma C Pulmonary congestion assessed by lung ultrasound in patients with severe aortic stenosis undergoing transcatheter aortic valve implantation: prevalence and prognostic implications. Eur J Heart Fail. (2024) 26(10):2107–17. 10.1002/ejhf.3365 39014551

[14] Généreux P Pibarot P Redfors B Mack MJ Makkar RR Jaber WA Staging classification of aortic stenosis based on the extent of cardiac damage. Eur Heart J. (2017) 38(45):3351–8. 10.1093/eurheartj/ehx381 29020232 PMC5837727

[15] von Elm E Altman DG Egger M Pocock SJ Gøtzsche PC Vandenbroucke JP. The strengthening the reporting of observational studies in epidemiology (STROBE) statement: guidelines for reporting observational studies. Lancet. (2007) 370(9596):1453–7. 10.1016/S0140-6736(07)61602-X 18064739

[16] Picano E Scali MC Ciampi Q Lichtenstein D. Lung ultrasound for the cardiologist. JACC Cardiovasc Imaging. (2018) 11(11):1692–705. 10.1016/j.jcmg.2018.06.023 30409330

[17] Stassen J Bax JJ. How to do lung ultrasound. Eur Heart J Cardiovasc Imaging. (2022) 23(4):447–9. 10.1093/ehjci/jeab241 34791145

[18] Kappetein AP Head SJ Généreux P Piazza N van Mieghem NM Blackstone EH Updated standardized endpoint definitions for transcatheter aortic valve implantation: the valve academic research consortium-2 consensus document (VARC-2). Eur J Cardiothorac Surg. (2012) 42(5):S45–60. 10.1093/ejcts/ezs533 23026738

[19] Platz E Lewis EF Uno H Peck J Pivetta E Merz AA Detection and prognostic value of pulmonary congestion by lung ultrasound in ambulatory heart failure patients. Eur Heart J. (2016) 37(15):1244–51. 10.1093/eurheartj/ehv745 26819225 PMC5006102

[20] Miglioranza MH Gargani L Sant’Anna RT Rover MM Martins VM Mantovani A Lung ultrasound for the evaluation of pulmonary congestion in outpatients: a comparison with clinical assessment, natriuretic peptides, and echocardiography. JACC Cardiovasc Imaging. (2013) 6(11):1141–51. 10.1016/j.jcmg.2013.08.004 24094830

[21] Araiza-Garaygordobil D Baeza-Herrera LA Gopar-Nieto R Solis-Jimenez F Cabello-López A Martinez-Amezcua P Pulmonary congestion assessed by lung ultrasound and cardiovascular outcomes in patients with ST-elevation myocardial infarction. Front Physiol. (2022) 13:1–8. 10.3389/fphys.2022.881626 PMC9127260 35620605

[22] Neves De Araujo G Beltrame R Pinheiro Machado G Luchese Custodio J Zimerman A Donelli Da Silveira A Comparison of admission lung ultrasound and left ventricular End-diastolic pressure in patients undergoing primary percutaneous coronary intervention. Circ Cardiovasc Imaging. (2021) 14(4):340–8. 10.1161/CIRCIMAGING.120.011641 33866795

